# Conceptual and pragmatic clinical implications of dichotomizing psychotropics as targeting physiology—physiomodal and pathology—pathomodal

**DOI:** 10.1093/ijnp/pyag006

**Published:** 2026-02-16

**Authors:** Michael Berk, Stephen M Stahl, Lakshmi Yatham, Janardhanan C Narayanaswamy, Seetal Dodd

**Affiliations:** Institute for Mental and Physical Health and Clinical Translation (IMPACT), School of Medicine, Deakin University, Geelong, VIC, Australia; Orygen, National Centre of Excellence in Youth Mental Health, Florey Institute for Neuroscience and Mental Health and Department of Psychiatry, University of Melbourne, Melbourne, VIC, Australia; Mental Health Drug and Alcohol Services, Barwon Health, University Hospital Geelong, Geelong, VIC, Australia; Department of Psychiatry and Neuroscience, University of California, Riverside, CA, United States; Department of Psychiatry, University of California San Diego, La Jolla, CA, United States; Institute of Mental Health, University of British Columbia, Vancouver, BC, Canada; Department of Psychiatry, Faculty of Medicine, University of British Columbia, Vancouver, BC, Canada; Institute for Mental and Physical Health and Clinical Translation (IMPACT), School of Medicine, Deakin University, Geelong, VIC, Australia; Mental Health Drug and Alcohol Services, Barwon Health, University Hospital Geelong, Geelong, VIC, Australia; Institute for Mental and Physical Health and Clinical Translation (IMPACT), School of Medicine, Deakin University, Geelong, VIC, Australia; Mental Health Drug and Alcohol Services, Barwon Health, University Hospital Geelong, Geelong, VIC, Australia; Centre for Youth Mental Health, University of Melbourne, Melbourne, VIC, Australia

**Keywords:** Psychotropics, psychopharmacology, classification, diagnosis, mental health, psychiatry

## Abstract

Conventional antidepressant, antipsychotics and mood stabilizing drugs share several characteristics. Firstly, they have a slow onset of action with benefits accruing late that is broadly mediated by long term homeostatic processes. Response generally predicts long term maintenance effects. They have little or no effect on the mental state of healthy individuals and can be conceptualized as targeting elements of pathophysiology. We can term them pathomodal. In contrast, psychotropic agents that are repurposed recreational drugs also share several effects. They have a rapid effect on mood states or perception and risk withdrawal symptoms that are generally the phenomenological mirror image of their acute effects and hence risks accrue late. Notwithstanding some heterogeneity, long term benefits are less clear than acute effects. Lastly, their effects are evident in all people who take them, and they can therefore be conceptualized as targeting elements of physiology. We can term them physiomodal. This categorization has several implications for both research design and clinical care.

**Key points**

- Conventional psychotropics share a slow onset of action with response generally predicting long term maintenance effects and they have little or no effect on the mental state of healthy individuals.
- They can be conceptualized as targeting elements of pathophysiology. We can term them pathomodal.
- In contrast, repurposed recreational drugs share a rapid effect on mood states or perception, a risk of withdrawal symptoms and long-term benefits that are less clear than acute effects. Lastly, their effects are evident in all people who take them.
- They can be conceptualized in targeting elements of physiology. We can term them physiomodal.

The arrival of the era of the repurposing of recreational drugs as potential therapeutic agents has led to a rapid transformation of the psychopharmacological landscape [1]. This prompts reconsideration of the class effects and classification of psychotropics. These novel interventions such as ketamine and psychedelics challenge the traditional paradigms that have classified psychotropics based on indication or neurotransmitter class. This categorization along the legacy of diagnostic-lines also has its shortcomings as the field moves towards transdiagnostic, dimensional, and circuit-based approaches.

Recreational drugs differ from conventional psychotropics in a substantial number of critical domains. Perhaps the most critical distinction is that psychotropic agents that are currently used in clinical practice such as lithium, anticonvulsants such as valproate and lamotrigine, diverse antidepressants, and antipsychotics have little to no effects in otherwise healthy people in contradistinction to their effects in those with psychiatric disorders. Lithium for example, when given to people without affective disorders has no material impact on mood. It even fails to make a substantive impact in mood instability in other psychiatric disorders like borderline personality disorder. The same is broadly true of antidepressants. Antipsychotics have no impact on non-psychotic dysfunctional thought patterns or misperceptions in otherwise healthy people. In contrast, as a class, recreational drugs have subjective and objective effects in all people who take them, with caveats regarding dose response and differential personal experiences. In this regard, opiates and benzodiazepine have more in common with recreational drugs.

Conventional psychotropics have other properties including a delayed onset of action with benefits generally slow to accrue. Effects of antidepressants and antipsychotics take several weeks to fully manifest. In counterpoint, response to acute treatment is generally predictive of response in the maintenance phase [2]. Homeostatic adaptation is thus critical to the mechanism of action of these agents.

In sharp contradistinction, rapid onset of subjective and objective effects is a class effects of recreational agents including psilocybin, ketamine, stimulants, opiates, cannabinoids and benzodiazepines. The flipside of recreational agents is the presence of withdrawal or discontinuation effects that generally reflect the opposite clinical profile to their acute effects. This may be an overgeneralization, but is evidenced for many drugs. Alcohol is relaxing and sleep inducing on acute use but associated with anxiety and insomnia on withdrawal [3]. Cannabis users endorse effects including improved sleep, calmness and relaxation, and increased creativity, while withdrawal symptoms include irritability, insomnia, and anxiety [4]. Stimulants decrease sleep need and increase energy and mood with acute use, but are associated with fatigue, lethargy, hypersomnia, depressed mood, and dysphoria on withdrawal [5]. Discontinuation of conventional agents prescribed to treat mental illness, presents risks of symptom re-emergence [6]. In sharp contradistinction to conventional psychotropics, homeostatic adaptation appears to be a pathway to risk and adverse events of most recreational agents.

## Pathomodal Agents

Conceptually then, traditional psychotropics could be described as “pathomodal” agents as they selectively target pathology and lack any psychotropic effects in otherwise healthy people. This seems to be a class effect of most conventional psychotropics. Adverse events are an exception in that they are present regardless of any underlying diagnosis. Pathomodal psychotropics are in broad terms thought to work putatively through downstream neuroadaptive regulatory processes such as changes in receptor sensitivity, synaptic plasticity, epigenetic changes and neurogenesis [7]. In this context, such a robust therapeutic response indicates strong effects on, and engagement with disorder-specific biological processes. The obvious caveat is that these specific processes are incompletely understood for almost all agents and disorders.

### Physiomodal Agents

Conversely, repurposed recreational drugs can be categorized as “physiomodal” agents as their effects are evident in all people using them and likely target physiological regulatory pathways. Their effects are rapidly evident across the spectrum from clearly unwell to clearly normal people and are independent of diagnosis. As a group they have a long history of use for recreational purposes precisely because they cause subjectively desirable short-term effects in almost all people in domains that may or may not overlap with the symptoms of psychiatric disorders. This appears to be a class effect of recreational drugs, including psychedelics, ketamine, stimulants, opiates, and benzodiazepines. While their effects may be therapeutically useful in certain specific situations (rapid anxiolysis with benzodiazepines, putative anti-suicidal effects of ketamine or trauma-related dysphoria reduction with MDMA), they share other properties including liability for abuse and dependence, rapid onset of action typically with the first dose, a variable (between individual and agent) degree of tachyphylaxis with sequential loss of efficacy and frequent withdrawal effects. The withdrawal effects vary from agent to agent, but in general are dysphoric and reflect the opposite effects of acute intoxication. For example, ketamine appears to have acute efficacy in reducing suicidal ideations, but there is preliminary evidence for a concerning signal, albeit based on limited data, of increased suicidality after discontinuation of a course of treatment [8]. Lithium discontinuation has also been associated with increased suicide but this is generally regarded as recurrence due to loss of maintenance protection [9].

The immediate mechanism of action of physiomodal agents is more likely to be through the direct receptor effects (N-methyl-D-aspartate [NMDA] antagonism for ketamine, GABA-A potentiation for benzodiazepines or serotonin 2A agonism for psychedelics) [10], noting that there is increasing evidence of subsequent changes for example regarding synaptic density after psilocybin treatment. Psilocybin has been termed a “plastogen,” catalyzing a swift onset of neuroplasticity which parallels with the onset of observed antidepressant action [11]. It needs to be acknowledged therefore that the boundary between pathomodal and physiomodal agents has a degree of fuzziness, in that the effects of some of conventional agents such as sedation are rapidly evident (although these could be due to off target effects such as on histamine), and some agents, possibly psilocybin, potentially having enduring effects beyond just physiological pathway engagement [11].

The pathomodal and physiomodal classification does not replace any existing nomenclature but offers a complementary and orthogonal axis, compatible with both existing classifications and the recent development of Neuroscience-based Nomenclature that uses neuroscience-based descriptors [12]. It cuts across neurotransmitter systems and provides a framework that has both clinical and research implications.

### Clinical Implications

So, what are the clinical implications of this conceptualization? While clearly not part of any accepted diagnostic system, response to prescribed treatment can reinforce both the prescriber and the consumers’ confidence in the allocated diagnosis, whereas lack of response can challenge the diagnosis, prompting consideration of alternatives. As an exemplar, in ADHD, the notionally desirable effects of stimulant medications that are evident in all people taking them on domains like sustained attention, motivation, focus and energy are subjectively desirable and may reinforce a putative diagnosis, relatively independent of whether threshold criteria are met.

It has also been argued that clear and dramatic response to a pathomodal psychotropic agent like lithium is possibly our best current biomarker capable of segregating individuals based on core disorder specific neurobiological processes. Despite an overt need, and extensive research, the promise of personalized psychiatry has not yet translated into clinical utility [13]. A bottom-up approach using treatment response as a biomarker intrinsically generates biologically relevant subtypes and should be a research priority. This is conceptually not possible for physiomodal agents.

The other clinical consideration is the epoch of required clinical vigilance. As an imperfect generalization, the beneficial effects of pathomodal agents are evident late, and maintenance effects among responders are predictable, whereas the beneficial effects of physiomodal agents occur early and problems like tachyphylaxis and withdrawal emerge late. Because of their slow onset of action, pathomodal agents require careful monitoring in the early phases of treatment to detect clinical change and risk, however once a person has responded and remains on maintenance therapy, risk attenuates, and the probability is that the sustained maintenance effects will be evident. The opposite may be true for physiomodal agents in that the time after discontinuation of treatment is the high-risk epoch and requires close monitoring. This is not normative clinical practice using traditional pathomodal agents, and clinical translation of physiomodal agents will require adaptation of the care model. Again, as a coarse generalization, while the acute benefits of pathomodal agents tend to predict maintenance efficacy, we do not yet know if those assumptions necessarily hold for physiomodal agents [1]. It remains unclear if physiomodal agents have robust long term maintenance effects, as most of the current research has explored acute effects.

### Research Implications

There are echoes in terms of clinical trial design which may need to be quite different. Again, conventional trial designs of pathomodal agents do not have routine post discontinuation follow up or observation periods as a part of standard protocols. However, these are intuitively more necessary in clinical trials of physiomodal agents, principally for the detection of withdrawal emergent risk and tight determination of long-term maintenance efficacy. As noted, pathomodal agents generally have discontinuation symptoms that are the opposite of the desired acute effects. This acknowledged generalization is evidenced for many drugs. Consequently, clinical trials of physiomodal agents should not copy the trial designs of pathomodal agents or they may fail to detect the long-term effects of these drugs. Post discontinuation follow up observations are likely needed for trials of physiomodal agents.

Research studies using pathomodal agents are best formulated in well-characterized clinical populations as therapeutic-effects are less likely to occur in healthy subjects. These trials can function as experiments to identify core-disease processes through treatment effect evaluations. In contrast, physiomodal agents can be examined across a wider participant range (healthy volunteers, at-risk individuals, and disease states) to assess processes that cut-across diagnostic boundaries such as emotion regulation or cognitive flexibility. Trials of pathomodal agents are suited to long-term disorder specific outcomes such as remission, relapse and functional recovery and inclusion of putative disorder specific biomarkers endpoints. Conversely, for physiomodal agents, outcome measures could focus more on transdiagnostic domains such as quality of life, dissociative experiences and suicidality.

Another difference between these categories is the trial duration – pathomodal agents generally need longer trial timelines and physiomodal agents allow for shorter trials (possibly enabling cross-over or within-subject designs). A major challenge in evaluating physiomodal agents is ensuring effective blinding in trials since the immediate effects of these agents are often associated with rapid and distinct subjective effects which can be profound and drive post randomization expectancy. Generally desirable subjective effects including elevated mood, anxiolysis, or perceptual changes can inject hope and optimism in people who have suffered enduring distress. Expectancy effects risk jeopardizing the internal validity of the studies with exaggeration of active effects by the expectancy responses [14]. This is in addition to the ethical and regulatory concerns due to potential abuse potential, often requiring stricter inclusion criteria and carefully regulated clinical environments.

A limitation of the physiomodal and pathomodal categories is that it suggests a sharp dichotomy when often the boundary between physiomodal and pathomodal action is likely semipermeable, at least for some agents. For example, with psychedelic drugs, it is unclear what pharmacological mechanism is responsible for the antidepressant action, rapid effect on the monoaminergic system through 5HT2A agonism or the slower neurotrophic effects. Possibly, both mechanisms are important. Psychedelics may promote dendritogenesis, synaptogenesis, neurogenesis, and expression of plasticity-related genes [15]. A broader implication could also be that this framework supports clinical reasoning (helping clinicians align the treatment expectations with drug-class properties), public communication (clarifying why certain medications are not subjectively felt but still effective) and harm reduction (establishing protocols for safely integrating physiomodal medications with psychotherapeutic frameworks - due to their cultural valence and subjective potency). Preclinical models can assist in clarifying many of these questions.

## Conclusions

In summary, dichotomizing psychotropics into physiomodal and pathomodal categories may have pragmatic utility in describing class effects with potential clinical and research implications. Future research should explore this framework across mental health disorders in longitudinal trajectories, incorporating biological correlates of class stratified treatment-response. The substantive clinical differences in the profiles of physiomodal and pathomodal agents can also serve as a framework for multiple clinical care considerations.
